# Systematic Review of PCR Proof of Parvovirus B19 Genomes in Endomyocardial Biopsies of Patients Presenting with Myocarditis or Dilated Cardiomyopathy

**DOI:** 10.3390/v11060566

**Published:** 2019-06-18

**Authors:** Angelos G. Rigopoulos, Bianca Klutt, Marios Matiakis, Athanasios Apostolou, Sophie Mavrogeni, Michel Noutsias

**Affiliations:** 1 Mid-German Heart Center, Department of Internal Medicine III (KIM-III), Division of Cardiology, Angiology and Intensive Medical Care, University Hospital Halle-Wittenberg, Mid-German Heart Center, Martin-Luther-University Halle-Wittenberg, Ernst-Grube-Straße 40, D-06120 Halle (Saale), Germany; angelos.rigopoulos@uk-halle.de (A.G.R.); marios.matiakis@uk-halle.de (M.M.); 2 Department of Anesthesiology, Helios Hospital Herzberg/Osterode, Dr.-Frössel-Allee, D-37412 Herzberg am Harz, Germany; Bianca.Klutt@harz.de; 3 School of Health Sciences, Faculty of Medicine, Department of Immunology & Histocompatibility, University of Thessaly, 41500 Larissa, Greece; thanosapostolou@gmail.com; 4 Onassis Cardiac Surgery Center, Department of Cardiology, and National Kapodistrian University of Athens, Leoforou Andrea Siggrou 356, Kallithea, 17674 Athens, Greece; sophie.mavrogeni@gmail.com

**Keywords:** parvovirus B19, B19V, erythrovirus, diagnosis, dilated cardiomyopathy, inflammatory cardiomyopathy, myocarditis, prognosis

## Abstract

Background: Diverse viral infections have been associated with myocarditis (MC) and dilated cardiomyopathy (DCM). In this meta-analysis, we summarize the published results on the association of parvovirus B19 (B19V) genomes with human MC/DCM versus controls. Methods: *n* = 197 publications referring to B19V and MC or DCM were retrieved using multiple PubMed search modes. Out of these, *n* = 29 publications met the inclusion criteria with data from prospective analyses on >10 unselected patients presenting with MC or DCM (dataset: MA01). Data retrieved simultaneously from both controls and MC/DCM patients were available from *n* = 8 from these publications (dataset: MA02). Results: In the dataset MA01 B19V genomes were detected in 42.6% of the endomyocardial biopsies (EMB) in this cohort by PCR. In the dataset MA02 comprising *n* = 638 subjects, there was no statistically significant different rate of B19V positivity in myocardial tissues comparing controls (mean: 38.8 + 24.1%) versus the MC/DCM-patients (45.5 + 24.3%; *p* = 0.58). There was also no statistical difference between the positivity rate of B19V genomes in myocardial tissues of MA01 (46.0 + 19.5%) and the two patient groups of MA02 (*p* > 0.05). Conclusions: This systematic review reveals that the mean rate of PCR detected B19V genomes in patients presenting with MC/DCM does not differ significantly from the findings in control myocardial tissues. These data imply pathogenetically insignificant latency of B19V genomes in a proportion of myocardial tissues, both in MC-/DCM-patients and in controls. More information (i.e., replicative status, viral protein expression) is pertinent to achieve a comprehensive workup of myocardial B19V infection.

## 1. Introduction

Acute myocarditis (AMC) and dilated cardiomyopathy (DCM), more specifically inflammatory cardiomyopathy (DCMi), are etiopathogenically linked entities. The highly diverse courses and long-term outcomes after AMC are substantially influenced by complex virus–host interactions at the post-acute/subacute phase after AMC [1,2].

In ca. 60% of endomyocardial biopsies (EMB) of the patients presenting with AMC or DCM, chronic intramyocardial inflammation, as detected by immunohistological quantification [3], and/or genomes of diverse viruses can be detected, consistent with the diagnosis of DCMi [4,5]. The immunohistological proof of DCMi is associated with adverse prognosis (mortality and indication for heart transplantation) [6].

Cardiac magnetic resonance (CMR) is helpful for the non-invasive detection of intramyocardial inflammation in the setting of MC and DCM [7,8]. However, CMR fails to specifically detect myocardial viral infection, including B19V [9]. Serology for anti-B19V IgG and IgM antibodies provides indications for a past or recent primary infection with B19V, however, it shows no statistically significant association with the detectability of B19V nucleic acids in myocardial tissues [10]. Thus, proof of viral genomes by polymerase chain reaction (PCR), including B19V, remains the mainstay for virological analyses in the context of MC/DCM/DCMi [1,11].

Various viruses have been associated with MC and DCM, with parvovirus B19 (B19V) having by far the highest prevalence (ca. 40%) [4,12,13]. The proof of viral genomes in EMB has been attributed as a new entity in the MOGE(S) classification (etiology: V—viral infection) [14]. This classification entity may also have implications for rational treatment strategies. Whereas disease specificity of enterovirus (EV) (i.e., Coxsackievirus) in this setting has been confirmed by meta-analysis for MC/DCM/DCMi patients [15], this relationship has not been established for B19V genomes yet. For B19V, a high prevalence, increasing with age, is well documented for many tested healthy tissues, and is referred to as the “bioportfolio” phenomenon [16]. Regarding B19V, no prognostic relevance has been elucidated for the polymerase chain reaction (PCR) proof of viral genomes in EMB [6]. In contrast to MC/DCM/DCMi patients with EV (i.e., Coxsackievirus) persistence [17], no beneficial effects have been achieved by antiviral interferon treatment for B19V positive DCMi patients [18]. These discrepancies might be due to a possible lack of disease specificity and of prognostic relevance of the mere PCR proof of B19V genomes in EMB from patients presenting with MC or DCM.

## 2. Materials and Methods

Electronic literature searches were carried out using Medline (via PubMed), Web of Science, the Cochrane Library, and Embase following the PRISMA (Preferred Reporting Items for Systematic Reviews and Meta-Analyses) statement [19]. The databases were searched by two independent reviewers on April 29th, 2014 by BK and MN. We combined the following keywords/ MeSH terms to identify the publications in several queries: “Parvovirus B19 OR B19V OR PVB19” AND “dilated cardiomyopathy”; AND “myocarditis”; AND “inflammatory cardiomyopathy”; AND “cardiomyopathy”. The literature search was conducted using EndNote Version X7.4 (Thomson Reuters, Eagan, MN, USA). We applied the following inclusion criteria: studies investigating >10 patients with clinically suspected myocarditis, DCM or DCMi, in whom EMB were obtained and processed for B19 genomes by PCR with defined protocols. We excluded publications referring to only animal experiments or in vitro experiments, human studies on <10 patients, case reports, congress reports, review articles, editorial letters, and publications written in languages other than English or German. Furthermore, we excluded publications reporting data from non-serially included patient groups, e.g., studies comparing pre-selected patient groups. The searches were reviewed by AGR. There were no discrepancies among the reviewers of the literature. We computed demographic data and the investigational results in a Microsoft Excel data table, which were then transferred to JMP statistical software (version 7.1; SAS Institute, Nancy, NC, USA).

### Statistical Analysis

Statistical analyses were performed using the software packages JMP and R (The R Project for Statistical Computing; version 3.2.0) with the packages “meta” and “metaphor” were employed for calculation of heterogeneity between the studies, forest plots, and funnel plots [20]. A probability value of *p* < 0.05 was considered statistically significant.

## 3. Results

**Figure 1:**
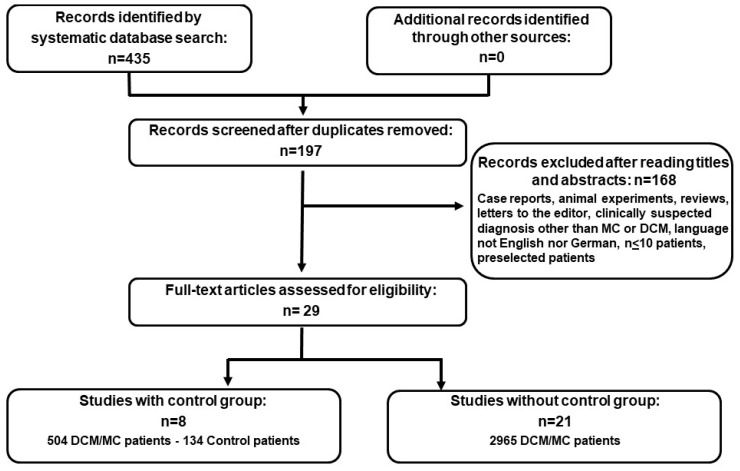
Flow chart for the selection of studies. The flow diagram shows the number of studies reviewed and included in the analysis as well as the number of the patients in the different study groups.

After exclusion of duplicates, *n* = 197 publications were found in the literature search. According to the inclusion criteria *n* = 29 publications with data from prospective analyses on 3424 subjects (dataset: MA01) were finally included in the synthesis. The study selection process is illustrated in the flow chart in Figure 1. The included publications without controls were *n* = 21, encompassing 2786 patients with clinically suspected MC or DCM. Data referring to controls (*n* = 134) and MC/DCM patients (*n* = 504) were available from *n* = 8 publications (dataset: MA02) (Table 1).

The demographic data of the *n* = 3,424 patients of the dataset MA01 were as follows: mean age: 49.7 ± 14.9 years; *n* = 2,301 men (67.2%). Echocardiographic data showed a mean left ventricular ejection fraction (LVEF) of 42.4 ± 13.2% and a mean left ventricular end-diastolic diameter (LVEDD) of 59.0 ± 8.1 mm. B19V genomes were detected by PCR in 42.6% of the EMB in this cohort. The mean reported B19V-IgG positivity was 51.1%.

A demonstration of the characteristics of patients belonging to the study group in studies without a control group (S1) as well as in studies with a control group (S2) is shown in Table 2. In the 21 studies without a control group there were 2786 patients with a mean age of 50.6 ± 14.7 years, and 63.9% were male. The echocardiographic parameters showed a mean LVEF of 45.2 ± 11.6% and mean LVEDD of 60.0 ± 9.0 mm. The detection of the B19V with PCR was seen in 46.0% of those cases.

In the eight studies with an incorporated control group (S2), 504 patients were in the study group. The mean age was 46.4 ± 12.2 years, 63.8% of patients were male, mean LVEF was 34.2 ± 9.0%, and the mean LVEDD was 63.8 ± 7.6 mm. The PCR positivity for B19V DNA was 45.5%. Those studies comprised 134 control patients (healthy myocardial tissue from donor hearts, patients with known coronary artery disease or arterial hypertension). These control patients were 61.9% male, with a mean age of 61.8 ± 8.6 years, mean echocardiographic LVEF 62.8 ± 8.8%, and mean LVEDD of 36.0 ± 2.0 mm. The PCR detected positivity for B19V was 37.5%.

### 3.1. Comparison of the Cardiac Parameters between Study and Control Groups

**Figure 2:**
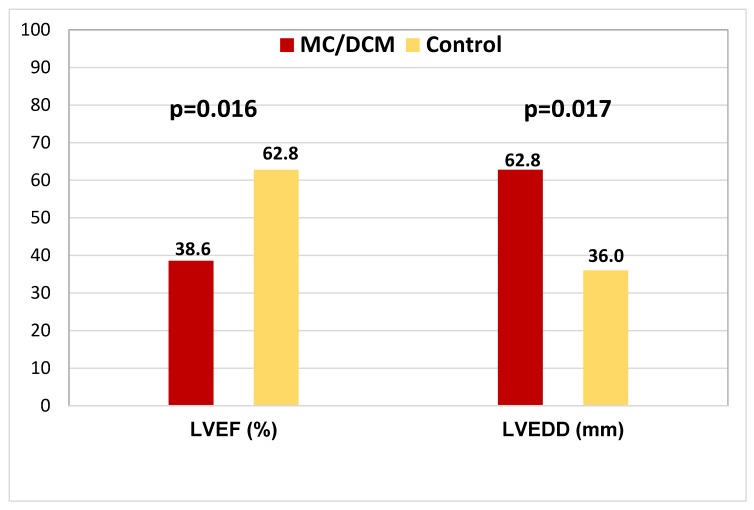
Comparing LVEF and LVEDD in the MC/DCM and in the control patients. The numbers on the bars represent the mean value. DCM: dilated cardiomyopathy; LVEF: left ventricular ejection fraction; LVEDD: left ventricular end-diastolic diameter; MC: myocarditis; DCM: dilated cardiomyopathy.

In comparison to the low LVEF of the DCM/MC patients (38.6 ± 10.0%), the LVEF of the healthy control patients, with 62.8 ± 8.8%, is significantly higher (*p* = 0.016) (Figure 2). The study patients also had a very high LVEDD (62.8 ± 7.5 mm) in comparison to the normal LVEDD (36 ± 2.0 mm) of the control patients (*p* = 0.007) (Figure 2).

### 3.2. Virus Detection Methods

In the studies included in the meta-analysis, three different methods were used to prove the presence of B19V: polymerase chain reaction (PCR), serology, and immunohistochemistry (IHC). The detection of B19V with PCR was used in 29 (100%) of the included studies.

Endomyocardial biopsies (EMB) were obtained from patients via a femoral venous (right ventricular) or arterial (left ventricular) access and snap frozen in liquid nitrogen. The detection of virus DNA was performed with the use of PCR (*n* = 19) or of nested PCR (nPCR; *n* = 15) employing special primers for the VP1-/VP2-/NS1-coding regions. The virus load was assessed by quantitative PCR, and was reported in a minor portion of the publications, which made meaningful statistical analyses impossible. In the 29 studies, B19V could be detected in 1688 (49.3%) patients with a mean PCR positivity rate of 45.9% ± 20.4% in the study group versus 38.7% ± 24.1% in the control group, respectively (*p* = 0.41).

Serology was performed in 10 studies comprising 844 patients and the presence of antibodies (anti-B19 V immunoglobulins) was demonstrated. A positive finding for B19V specific antibodies was reported in 401 (47.5%) of the patients, with 46.1% ± 36.4% in the study group and 35.8% ± 37.8% in the control group (*p* = 0.73). The detection of IgM antibodies was reported in 18 (4.5%) of the patients, and of IgG antibodies in 379 (94.5%) of the patients, respectively. There was no statistical difference regarding the detectability of B19V-specific IgG, with 58.7% ± 28.7% in the study group versus 47.3% ± 21.5% in the control group (*p* = 0.63). Furthermore, no statistical difference was calculated for the reported B19V-specific IgM, with 2.8% ± 4.8% in the study group versus 1.8% ± 2.5% in the control group (*p* = 0.79).

For the immunohistological detection of B19V proteins (VP1 and VP2), cryosections from frozen EMB were examined in 18 studies and the B19V protein expression was reported in 43.1% of MC-/DCM-specimens. No data were available for the immunohistological detection of B19V proteins in EMB from control patients.

### 3.3. Distribution of Viruses in Patients with DCM/MC and Control Patients

In all study patients apart from B19V, which was detected in 1688 (49.3%) patients, other viruses were also detected in a smaller percentage: enterovirus, human herpesvirus 6 (HHV-6), adenovirus (ADV), Epstein-Barr virus (EBV), and cytomegalovirus (CMV) (Table 3). Multiple infections with simultaneous detection of various viral genomes was reported in *n* = 163 (12.3%) patients.

### 3.4. PCR Proof of B19V Genomes Comparing Patients with DCM/MC and Controls

The main attention of this meta-analysis was focused on the question, if a B19V infection can be a risk factor for DCM/MC or if there is no difference in comparison to donor hearts. In order to clarify this, a direct comparison between DCM/MC patients and healthy donor hearts by using PCR was performed in the MA02 cohort comprising *n* = 638 subjects. There was no statistically significant different rate of B19V positivity in myocardial tissues comparing controls (mean: 38.8 ± 24.1%) versus the MC/DCM-patients (45.5 ± 24.3%; *p* = 0.58). There was also no statistical difference between the positivity rate of B19V genomes in myocardial tissues of MA01 (46.0 ± 19.5%) and the two patient groups of MA02 (*p* > 0.05).

**Figure 3:**
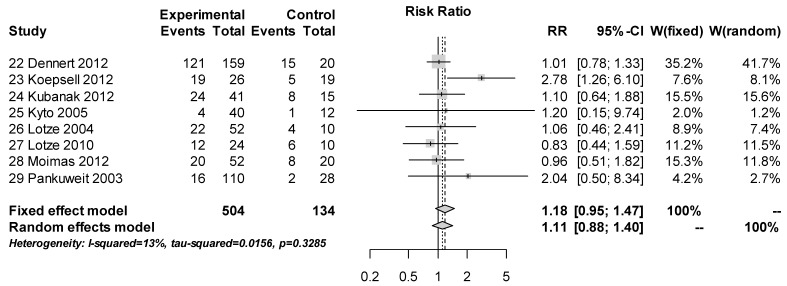
Plot of the *n* = 8 studies with control group. The mean relative risk (RR) is represented with the grey diamond and the dotted vertical line. The grey squares show the RR for the individual studies, the horizontal lines show the corresponding 95% confidence intervals. Experimental: study group, Control: control group, Events: sum of events, Total: number of patients of the corresponding group, RR: relative risk.

The forest plot of the eight studies with a control group shows the comparison of B19V detection with PCR in DCM/MC patients and control patients (Figure 3). No significantly higher detection rate of B19V in the study group was shown in comparison to the control groups of these eight studies (*p* = 0.3285)

## 4. Discussion

In patients presenting with MC or DCM, EMB can be performed as a diagnostic procedure [1,44]. Apart from the histological and immunohistological diagnosis, PCR detection of virus genomes is recommended [11]. In this context, B19V genomes are most frequently detected compared with other viral genomes studied [13,21,45].

For the enterovirus/Coxsackievirus B3 there is a causative association between detection of virus genome and appearance of myocardial disease. Apart from the experimental confirmation in murine models, a meta-analysis has also shown the association between enterovirus and MC/DCM compared to controls; a prognostic as well as therapeutic importance has also been shown [15,17,46]. However, such an association has not been proven convincingly for B19V in MC/DCM. The main aim of our study was to systematically review the published studies focusing on this topic, and to perform a meta-analysis of the available data. Similar detection rates with B19V genomes have been documented in various regions of the world, including Europe, North America, and Asia [47]. The range of seropositivity for B19V rises substantially from fetal to advanced age. In Germany, seropositivity for B19V extends from 20.4% in children up to 79.1% in people over 65 years of age [48]. Similar data could also be shown in England/Wales (21%–75%), Belgium (74%), and Italy (79%) [49]. The transmission of B19V occurs through droplets, blood, or close body contact. After seroconversion, lifelong seropositivity ensues. The often asymptomatic course after first B19 infection facilitates the spread of the virus. B19V usually persists mostly lifelong in various tissues, a phenomenon also known as “bioportfolio” [16,47]. For tonsillar tissues, Pyöriä et al. have recently identified B cells as the main cell type of B19V genome persistence [50]. This investigation also supports the maintenance of pathogen-specific humoral immune responses as a consequence of B-cell long-term survival. Thus, the frequent detection of B19V genomes in EMB of DCM/MC patients raises the question of pathogenic relevance, since it might be in parts due to lifelong persistence of B19V DNA in terms of a “bioportfolio” effect as well [51,52].

### 4.1. PCR Diagnosis of B19V

In the 21 studies without a control group, which assessed 2786 DCM/MC patients, B19V genomes were proven by PCR in 50.3% of the patients. A similar rate of B19V proof of 56.9% was also reported in the DCM/MC patients of the eight studies which included a control group. In total, in the 29 studies including 3290 DCM/MC patients, B19V genomes were detected by PCR in EMB in 49.3% of the patients. In the control group, detection of B19V by PCR was reported in 37.5% of EMB specimens, being not significantly different to the findings in control patients (*p* = 0.509). These data imply pathogenetically insignificant latency of B19V genomes in a proportion of myocardial tissues of both MC-/DCM-patients and controls. Thus, PCR evidence of B19V genomes is not sufficient to indicate a disease relationship between B19V and MC/DCM [51,52]. These data may be compatible with the “bioportfolio” phenomenon, known for the PCR detection of B19V genomes in other human tissues [16]. More information (i.e., replicative status, viral protein expression) is pertinent to achieve a comprehensive workup of myocardial B19V infection [28,29,42,52]. The lack of such additional information could be a pivotal reason for the neutral trial result of an anti-viral interferon treatment study in MC/DCM associated with B19V, which was only based on the PCR proven B19V genome in EMB [18].

### 4.2. Factors Potentially Influencing EMB Diagnosis

The 29 studies included in this meta-analysis have been performed in different centers with different assessment methods. The heterogeneity of the inclusion criteria of the 29 studies as well as of the diagnostic methods and the measured results might have contributed to heterogeneity. The advances in virus load quantification, differentiation of genotypes and of viral replications status are additional methodological issues which cannot be unified in all studies, since the technical evolution of these methods was incremental over time. Hence, the first investigations did not address these detailed issues. It can be assumed that there were differences in the size and quantity of EMB tissue specimens among the 29 studies, as well as multiple sources of methodological differences regarding DNA extraction and PCR protocols. Depending on the individual center, different primers and probes were used for the PCR. Besides, the number of obtained EMB samples per patient, from which DNA is extracted, varied significantly among the different studies from 1 to 6 EMB for evaluation of viral genomes [12,13,22]. Another important issue may be the sampling error, which is well known to impair the diagnostic accuracy of histological EMB evaluation [53], but is not precisely known for the virological analyses of EMB [54]. Finally, the problem of the preferred ventricle for the obtainment of EMB in the context of myocarditis or DCM has not been solved yet [7,55]. Another important factor is the timing of EMB in the natural course of the disease, with higher amounts of virus load in the acute compared to the chronic phase [45,56]. Additionally, the prognostic relevance of differentiating the B19V genotype is incompletely understood [30,36]. Taken together, these data highlight the importance of standardized, uniform methodological approaches for the proof of relevant myocardial B19V infections.

### 4.3. Control Group

The data on myocardial samples from the control group are extremely important, since these are the only available data comparing B19V infections in non-MC and non-DCM patients. Nonetheless, the ideal myocardial samples would have been EMB from cardiovascular healthy, age- and sex-matched controls, which however is not possible due to obvious ethical constraints. A further issue is the not well-standardized sampling region among the eight studies.

### 4.4. Prognostic Relevance of PCR Proven B19V Genomes in the Endomyocardium

In a prospective single center study with clinically suspected myocarditis, the PCR proof of viral genomes in EMB, including B19V, was not associated with prognosis, as opposed to the adverse outcome in patients with immunohistological proof of intramyocardial inflammation [6]. The adverse prognostic impact of intramyocardial inflammation was also confirmed in investigations focusing on cardiac magnetic resonance (CMR) based detection of late gadolinium enhancement (LGE) in patients presenting with both MC and DCM [57,58]. Further publications did not confirm an adverse impact of B19V genomes in EMB of patients with clinically suspected MC or DCM [27,35]. Only in one study, focusing on highly selected DCM patients with diverse courses of spontaneous viral elimination versus viral persistence, a potential prognostic impact was reported for viral genomes persisting over 6 months of follow up EMB investigations, including B19V, either as monoinfection or as part of multiple viral genomes present in EMB [5]. These insights were not confirmed in a comparable analysis [59]. So far, we are lacking detailed data on comprehensive B19V investigations including the kinetics of viral loads, the differentiation of the B19V replication status, the B19V genotypes, the B19V protein expression pattern, and the cellular and humoral antiviral immune responses in selected patients with biologically relevant myocardial B19V infections.

### 4.5. Future Management Strategies

Evidence based management strategies of MC/DCM patients are based on general heart failure guidelines [60]. In rare cases of giant cell or eosinophilic myocarditis, immunosuppressive treatment has a class I indication for the improvement of the outcome [44]. For the time being, there is no widely accepted, evidence based anti-viral or pathogen directed therapy for a viral MC/DCM [18,61]. Antiviral interferon-beta treatment has shown positive clinical and prognostic effects in enteroviral/Coxsackievirus B persistence in single center and multicenter studies, including some evidence for effective viral elimination, however, this antiviral treatment has not proved as effective in B19V associated MC/DCM [17,61]. Selection of B19V positive patients with high B19V loads might be a relevant approach to identify subgroups of B19V patients who might benefit from immunomodulatory treatment [62]. Comprehensive, standardized diagnostic differentiation of MC/DCM patients with endomyocardial B19V infections with biological and prognostic relevance (possibly including viral replication, myocardial B19V protein expression, and ongoing active cellular and/or humoral anti-B19V immune response) might be a key approach for both an updated diagnostic classification of B19V associated viral cardiomyopathy [14], and a more meaningful selection of candidates for future antiviral immunomodulatory treatment trials.

### 4.6. Limitations of the Study

All potential limitation issues known for meta-analyses also apply to this study [63]. This meta-analysis enables a standardized aspect of the available evidence of virus diagnosis in DCM/MC patients; it portrays no general recommendation for the further procedure in a viral infection coexistent with myocardial disease. For the evaluation of individual measured data from the studies it was not always possible, because of missing statements about the results, to show mean values. For the determined virus load of B19V using PCR no mean value could be reported, because in the few studies in which the virus load was reported, either an individual value or a total measurement range was given. For the assessment of B19V load by using PCR in the control group no mean value could be calculated, as only two studies reported such values. An overview of the values of B19V loads in EMB from patients versus healthy study participants would be of paramount importance, because significant differences between MC and DCM patients were reported [45]. Nevertheless, it must be stated, that the comparison of patient groups in this publication was merely based on the presumed clinical diagnosis that was reported to the pathology institute when sending in the EMB samples. Thus, a reliable verification of the clinical parameters in this publication is impossible, and the evaluation of these data cannot be regarded as representative.

## 5. Conclusions

This meta-analysis shows that the mere PCR proof of B19V genomes in EMB has no significant association with the clinical diagnosis of MC/DCM, since this finding is equally present in control hearts. The lack of disease specificity is compatible with the “bioportfolio” phenomenon known for other non-cardiac organs [16]. The appreciation of these insights improve our understanding of the missing significant clinical effects of immunomodulatory strategies in MC-/DCM-patients associated with B19V genomes EMB proven by PCR. In order to identify biologically relevant B19V myocardial infections, additional characteristics of a B19V infection and of the anti-B19V immune response might be helpful, such as the B19V virus load, the differentiation of B19V genotype, the B19V replication status, the characterization of myocardial B19V protein expression patterns, and the differentiation between an active humoral and cellular anti-B19V response versus the pathogenetically insignificant latent persistence of B19V genomes [28,29,52,56]. This comprehensive, standardized characterization could lead to the development of specific features for the selection of well characterized B19V positive MC-/DCM-patients who may ultimately profit from tailored anti-viral immunomodulatory treatment strategies [51].

## Figures and Tables

**Table 1:** Studies included in the meta-analysis.

Study Code	First Author	Year	Journal; Citation	Total Number of Study Subjects (n)	Number of Patients in the MC-/DCM-Group (n)	Number of Patients in the Control Group (n)
*01*	Kuhl	2003	Circulation; [21]	24	24	
*02*	Pankuweit	2003	Hum Pathol; [12]	138	110	28
*03*	Lotze	2004	Med Microbiol Immunol; [22]	62	52	10
*04*	Mahrholdt	2004	Circulation; [7]	32	32	
*05*	Vallbracht	2004	Circulation; [23]	124	124	
*06*	Kuhl	2005	Circulation; [13]	245	245	
*07*	Kuhl	2005	Circulation; [5]	172	172	
*08*	Kyto	2005	Clin Infect Dis; [24]	52	40	12
*09*	Tschöpe	2005	Circulation; [25]	70	70	
*10*	Mahrholdt	2006	Circulation; [26]	128	128	
*11*	Kuethe	2007	Am Heart J; [27]	197	197	
*12*	Escher	2008	Med Sci Monit; [28]	30	30	
*13*	Escher	2008	Med Sci Monit; [29]	62	62	
*14*	Kuhl	2008	J Med Virol; [30]	317	317	
*15*	Schenk	2008	J Clin Microbiol; [31]	69	69	
*16*	Yilmaz	2008	Heart; [32]	85	85	
*17*	Zimmermann	2009	Basic Res Cardiol; [33]	66	66	
*18*	Moulik	2010	J Am Coll Cardiol; [34]	94	94	
*19*	Zimmermann	2010	J Card Fail; [18]	110	110	
*20*	Lotze	2010	J Med Virol; [35]	34	24	10
*21*	Mahfoud	2011	Eur Heart J; [10]	124	124	
*22*	Ruppert	2011	J Med Virol; [36]	139	139	
*23*	Stewart	2011	Circ Heart Fail; [37]	100	100	
*24*	Dennert	2012	Clin Vaccine Immunol; [38]	179	159	20
*25*	Koepsell	2012	Cardiovasc Pathol; [39]	45	26	19
*26*	Kubanek	2012	Eur J Heart Fail; [40]	56	41	15
*27*	Moimas	2012	Heart Lung Circ; [41]	72	52	20
*28*	Kuhl	2013	Basic Res Cardiol; [42]	537	537	
*29*	Miranda	2014	Cardiol Young; [43]	61	61	

The included studies are listed in chronological order according to publication year and demonstrated with the study code for the meta-analysis and the name of the first author. The number of MC/DCM patients equals the total number of study subjects in the studies without a control group. DCM: dilated cardiomyopathy; MC: myocarditis.

**Table 2:** Characteristics of the subgroups of the study group.

	S1	S2
Number of patients (DCM/MC) (n=)	2786	504
Male [%]	63.92	63.76
Age, mean ± SD [years]	50.62 ± 14.65	46.37 ± 12.2
LVEF, mean ± SD [%]	45.17 ± 11.64	34.18 ± 8.96
LVEDD, mean ± SD [mm]	59.95 ± 8.98	63.83 ± 7.56
PCR positive for B19V [%]	49.6	47.2

DCM: dilated cardiomyopathy, LVEDD: left ventricular end-diastolic diameter, LVEF: left ventricular ejection fraction, MC: myocarditis, PCR: polymerase chain reaction, S1: studies without control group, S2: studies with control group.

**Table 3:** Distribution of viruses in MC-/DCM-patients and in control patients.

Virus	n (%)
Parvovirus B19 (B19V)	1688 (46.85%)
Enterovirus (EV)	203 (5.36%)
Human Herpes virus-6 (HHV-6)	176 (4.88%)
Adenovirus (EDV)	44 (1.22%)
Epstein-Barr virus (EBV)	30 (0.83%)
Cytomegalovirus (CMV)	28 (0.77%)

## Abbreviations

B19V	parvovirus B19
CMR	cardiac magnetic resonance
DCM	dilated cardiomyopathy
DCMi	inflammatory cardiomyopathy
EMB	endomyocardial biopsy or biopsies
EV	enterovirus: enteroviral
IHC	immunohistochemical
LGE	late gadolinium enhancement
LVEF	left ventricular ejection fraction
LVEDD	left ventricular end-diastolic diameter
MC	myocarditis
PCR	polymerase chain reaction
